# The Role of Licorice Chalcones as Molecular Genes and Signaling Pathways Modulator—A Review of Experimental Implications for Nicotine-Induced Non-Small Cell Lung Cancer Treatment

**DOI:** 10.3390/cimb46060352

**Published:** 2024-06-13

**Authors:** Naser A. Alsharairi

**Affiliations:** Heart, Mind and Body Research Group, Griffith University, Gold Coast, QLD 4222, Australia; naser.alsharairi@gmail.com

**Keywords:** non-small cell lung cancer, *Glycyrrhiza*, licorice, chalcones, isoliquiritigenin, licochalcone, echinatin, nicotine, experimental studies

## Abstract

Lung cancer (LC) represents the leading cause of global cancer deaths, with cigarette smoking being considered a major risk factor. Nicotine is a major hazardous compound in cigarette smoke (CS), which stimulates LC progression and non-small cell lung cancer (NSCLC) specifically through activation of the nicotinic acetylcholine receptor (α7nAChR)-mediated cell-signaling pathways and molecular genes involved in proliferation, angiogenesis, and metastasis. Chalcones (CHs) and their derivatives are intermediate plant metabolites involved in flavonol biosynthesis. Isoliquiritigenin (ILTG), licochalcone A–E (LicoA–E), and echinatin (ECH) are the most common natural CHs isolated from the root of *Glycyrrhiza* (also known as licorice). In vitro and/or vivo experiments have shown that licorice CHs treatment exhibits a range of pharmacological effects, including antioxidant, anti-inflammatory, and anticancer effects. Despite advances in NSCLC treatment, the mechanisms of licorice CHs in nicotine-induced NSCLC treatment remain unknown. Therefore, the aim of this paper is to review experimental studies through the PubMed/Medline database that reveal the effects of licorice CHs and their potential mechanisms in nicotine-induced NSCLC treatment.

## 1. Introduction

Globally, lung cancer (LC) is regarded as the most common malignant tumor causing death, with tobacco smoking being considered the greatest risk factor [1,2,3]. Non-small cell lung cancer (NSCLC) and small cell lung cancer (SCLC) are the two major types associated with tobacco smoking, with NSCLC comprising 85% of all LC cases while SCLC represents the remaining 15% [3]. NSCLC is further categorized into large cell carcinoma, adenocarcinoma, and squamous subtypes [3].

**Figure 1:**
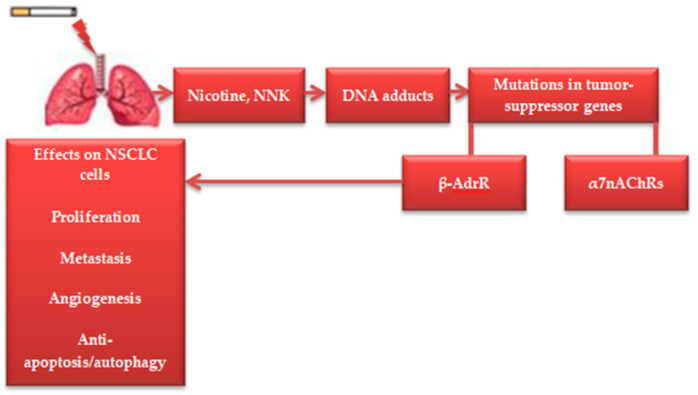
Mechanisms of nicotine in NSCLC carcinogenesis.

Tobacco smoke consists of multiple chemicals identified as carcinogens, such as “4-(Methylnitrosamino)-1-(3-pyridyl)-1-butanone” (NNK) and “*N*-Nitrosonornicotine” (NNN), which contribute to NSCLC risk [4]. While nicotine is not a carcinogen, it could be the cause of the increasing NSCLC risk in smokers [5]. Both nitrosamines (NNK, NNN) and nicotine demonstrated significant induction of the nicotinic acetylcholine receptor (α7nAChRs) and beta-adrenergic receptor (β-AdrR) expressed in several brain areas (e.g., hypothalamus), which could, in turn, promote tumor growth, proliferation, angiogenesis, and metastasis in NSCLC cells [4,5,6]. The mechanisms through which nicotine causes NSCLC have been widely described in the literature [6,7]. Figure 1 shows the mechanisms of nicotine in NSCLC progression.

Chalcones (CHs), also termed 1,3-diphenyl-2-propen-1-ones, are plant metabolites that serve as precursors for isoflavonoids and flavonoids biosynthesis in edible and medicinal plants [8]. CHs also act as positive allosteric modulators (PAMs) of human α7nAChR channels polyhydroxy-substituted chalcone and diphenyl propanones [9]. CHs have a ketoethylenic moiety in their structure and consist of benzene rings (A and B) that are connected through a three-carbon α, β-unsaturated carbonyl system. Moreover, CHs possess a two-fold conjugate bond and exhibit different π-electron delocalization on both rings [8,10,11].

**Figure 2:**
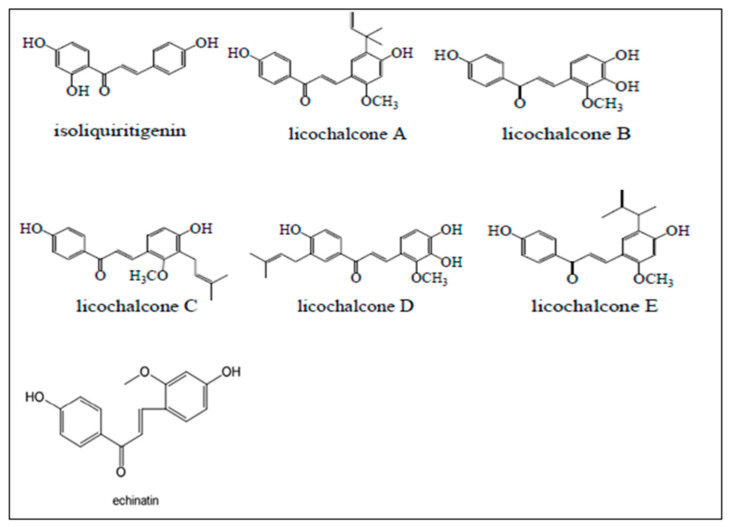
Chemical structures of licorice CHs [23,24].

Natural CHs have been widely studied and often exhibit anticancer, antidiabetic, anti-inflammatory, antioxidant, and neuroprotective activities [12,13,14,15]. Natural CHs are categorized based on the type of subtraction into methoxy, amino, alkyl, aryl, and hydroxy CHs [16]. Isoliquiritigenin (ILTG), licochalcones A–E (LicoA–E), and echinatin (ECH) are natural hydroxy-methoxy CHs, mainly isolated from the *Glycyrrhiza* genus (also known as licorice), which belongs to the leguminosae/Fabaceae family, which consists of several species, where *G. glabra*, *G. uralensis*, and *G. inflate* are the most common ones [17]. These CHs exert anti-inflammatory, anti-angiogenesis, apoptosis, and cell cycle arrest activities in cancer cells through a wide range of molecular mechanisms such as cyclin-dependent kinase 1 (CDK1), proliferating cell nuclear antigen (PCNA), and nuclear transcription factor kappaB (NFκB) [17,18,19,20,21,22]. The chemical structures of natural CHs derived from *Glycyrrhiza* (ILTG, LicoA–E, and ECH) are shown in Figure 2.

Licorice CHs have been regarded as safe, but the main issue lies with the range of safe doses in treatment [18]. There is still a research gap on whether licorice ILTG for use in treating cancer is safe and nontoxic [25]. However, in vitro and in vivo experimental evidence has suggested that licorice LicoA and ILTG counteract the toxicity of chemotherapeutic drugs, along with enhancing anticancer activity [19]. A few in vivo experiments in rats showed that the bioavailability of ILTG is frequently low, which is attributed to the disappearance and/or rapid formation of ILTG metabolites in the liver and small intestine [26,27]. The administration of ILTG to rats at a dose of 20 mg/kg led to low absorption efficiencies and a rapid disappearance of ILTG in different tissues (liver, heart, lung, and kidney) [28]. An experiment has revealed a very small amount of the ILTG metabolite isoliquiritin apioside absorbed by passive diffusion in the intestinal Caco-2 cell monolayer [29]. It was reported that the absorbed LicoA and ECH in rat plasma were 3.3% and 6.81%, respectively, after oral (15 mg/kg) and intravenous (5 mg/kg) administeration of LicoA and ECH [30,31].

Research evidence suggests that licorice-derived flavonoids exert autophagic effects against NSCLC cells in vivo through downregulating the CDK4-cyclin D1 signaling pathway [32]. Natural flavonoids, including flavones, anthocyanins, proanthocyanidins, and quercetin derived from medicinal plants (e.g., *Scutellaria baicalensis*, *Vaccinium macrocarpon*, *Myrica rubra*, *Rhododendron formosanum*, *Ginkgo biloba*, *Polygonum aviculare*), are thought to be effective in nicotine-induced NSCLC treatment due to their anti-proliferative, anti-angiogenesis, anti-inflammatory, and apoptotic/autophagic properties [33,34,35]. However, the effects and mechanisms of licorice CHs in nicotine-induced NSCLC treatment remain largely unknown. Thus, this is the first review to cover experimental studies summarizing the therapeutic effects of licorice CHs in nicotine-induced NSCLC and exploring the mechanisms of action that underlie their effects.

## 2. Methods

A literature search was performed to identify articles in the PubMed/Medline database until 15 May 2024. The search was conducted using the “AND” Boolean function to combine the following keywords: “NSCLC”, “*Glycyrrhiza*”, “licorice”, “ILTG”, “Lico”, and “ECH”. Experimental studies published in English on the effects and/or mechanisms of licorice CHs in nicotine-induced NSCLC treatment were included. Studies were excluded if they focused on emphysema, as it has different cellular and/or molecular mechanisms underlying nicotine-induced lung injury compared to NSCLC [36]. The search identified 24 experimental studies that met the search criteria, which included 19 in vitro, 1 in vivo, and 4 mixed (in vitro and in vivo).

## 3. Licorice Isoliquiritigenin in Nicotine-Induced NSCLC Treatment

Among CHs, ILTG (4,2′,4′-trihydroxychalcone) is considered the most active one, which acts as a PAM for α7nAChR due to its ability to facilitate agonist-induced activation of α7 choline receptors without interacting with other subtypes of selective nAChR agonists [9]. The PAM activity of ILTG results from the substitution of the hydroxyl (OH) groups from position 2 to 4 of ring B, combined with the presence of a hydroxy substitution at the 2′ or 4′ position of ring A [9]. Treatment with ILTG showed inhibition of nicotine-induced inflammasome activation in the brain of female Sprague-Dawley rats [37]. It has been reported that amyloid-beta (Aβ)_1–42_ aggregation could be inhibited by ILTG in vitro, which may be due to the presence of a 4-substituted side chain in the A ring of ILTG, thereby producing π-π and hydrophobic bonds with Aβ_1–42_ [38]. ILTG was also found to attenuate Aβ_42_-induced oxidative stress in microglia by reducing the production of inflammatory cytokines and nitric oxide (NO) through the downregulation of NFκB and the upregulation of nuclear factor erythroid-2 related factor 2 (Nrf2) signaling pathways [39]. Activation of the α7nAChR-mediated signaling pathways and molecular genes involved in NSCLC cell proliferation and anti-apoptosis were found to aggregate Aβ_40_ and Aβ_42_ levels, which inhibit protein 53 (p53)-induced apoptosis, leading to the upregulation of the phosphatidylinositol-3 kinase/threonine kinase/NFκB (PI3K/Akt/NFκB) signaling pathway and metalloproteinase 2/9 (MMP2/9) expression [40]. Therefore, ILTG might have anti-cancer effects by regulating cellular processes in NSCLC cells through downregulation of the α7nAChR-mediated signaling pathways and gene expression involved in Aβ aggregation.

ILTG also has the potential to inhibit monoamine oxidase (MAO) in vitro by interacting with active site residues at dopamine and vasopressin receptors, thereby reducing neuronal disorders [41]. MAO promotes metastatic potential in NCI-A549 NSCLC cells in such a way that it increases migration and reactive oxygen species (ROS) production through the activation of multiple signaling pathways, including signal transducer and activator of transcription (STAT1/3/6), cAMP-responsive element-binding protein (CREP), early growth response 1 (EGR1), and peroxisome proliferator-activated receptor γ (PPARγ) [42]. MAO is indeed highly expressed in NSCLC cells, and MAO knockout obviously inhibits human papillomavirus (HPV)-16 E7 oncoprotein-induced tumor growth, hypoxia-inducible factor-1 (HIF-1α) protein accumulation, ROS production, and the expression of epithelial-mesenchymal transition (EMT)-associated markers in NCI-H460 NSCLC cells [43]. MAO inhibitors G10 and G11 have been reported to exhibit high inhibitory effects on paclitaxel-resistant NCI-A549/H460 NSCLC cell proliferation, growth, and metastasis [44,45], through the downregulation of the Akt/HIF-1α signaling pathway and MMP2/p21 expression [45]. Thus, ILTG may have significant inhibitory effects on MAO-induced migration, proliferation, growth, and metastasis of NSCLC cells through the downregulation of a7nAChR-mediated signaling pathways and molecular genes.

ILTG has shown in a few experiments its therapeutic potential in nicotine-induced NSCLC by inhibiting proliferation, invasion, migration, and tumor growth, along with the induction of apoptosis and autophagy via modulating the α7nAChR-mediated signaling pathways and/or molecular genes. Treatment with ILTG resulted in the inhibition of proliferation, invasion, and migration by inducing apoptosis in NSCLC cells in vitro at a concentration of 20 μM. This was demonstrated by activating the expression of pro-apoptosis-associated genes, and inhibiting the expression of anti-apoptosis, proliferation, invasion, and migration-associated genes, as well as the PI3K/Akt signaling pathway [46]. In vitro experiments showed anti-proliferative and apoptotic effects on NSCLC cells following treatment with 20 and 40 μmol/L ILTG by blocking the cell cycle at the G_1_ phase via upregulating expression of apoptosis and cell-cycle-associated genes [47]. ILTG at a concentration of 40 μM induces proliferation inhibition and G_2_/M cell cycle arrest in NSCLC cells through a mechanism that involves upregulating cell cycle-related genes [48]. The combinational treatment of NSCLC cells with ILTG and flavonoid glycosides (liquiritin, isoliquiritin) extracted from *G. uralensis* resulted in the induction of apoptosis, cytotoxicity, and cell cycle arrest at the G_2_/M phase in vitro [49]. ILTG was found to be effective in inhibiting tumor growth of NSCLC cells in vitro when treated at a concentration of 50 μg/mL by suppressing the localization of β-catenin to the nucleus [50]. It has been shown that ILTG inhibits the proliferation, invasion, and migration of NSCLC cells, with significant cytotoxicity observed at different concentrations in vitro. This inhibition was mediated by downregulating oncofetal IGF2 mRNA-binding protein 3 (IGF2BP3) expression, which promoted the mRNA stability of twist family bHLH transcription factor 1 (TWIST1) [51]. An in vitro experiment on NSCLC cells showed that apoptosis and cytotoxicity were significantly enhanced after treatment with ILTG nanosuspension in comparison to pure ILTG at different concentrations. However, the mechanisms behind these effects have not been elucidated [52].

In vitro and in vivo experiments reported inhibition of NSCLC cell migration, invasion, and metastasis when treated with ILTG at a concentration of 10 μM in a mechanism possibly involving blocked cytoskeleton reorganization and focal adhesion assembly through reducing the abundance of tyrosine (Tyr)-phosphorylated non-receptor tyrosine kinase (Src) and focal adhesion kinase (FAK) [53]. ILTG was also found to significantly induce apoptosis in tyrosine kinase inhibitor (TKI)-resistant and sensitive NSCLC cells both in vitro and in vivo at a concentration of 40 μM by targeting mutant/wild type epidermal growth factor receptor (EGFR) through downregulating Akt and extracellular signal-regulated kinase (ERK)1/2 signaling pathways, B-cell lymphoma-2 (Bcl-2) expression, and upregulating poly ADP ribose polymerase (PARP) cleavage, B-cell chronic lymphocytic leukemia-lymphoma like 11 gene (Bim), and caspase 3 expression [54].

**Figure 3:**
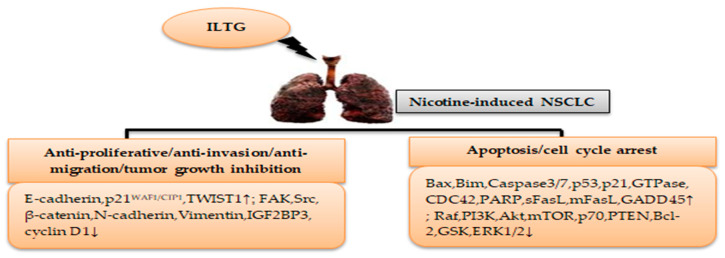
Mechanisms of ILTG in nicotine-induced NSCLC treatment. (↓) decrease, (↑) increase.

Table 1 and Figure 3 highlight the therapeutic effects and mechanisms of licorice ILTG in nicotine-induced NSCLC.

## 4. Licorice Licochalcone in Nicotine-Induced NSCLC Treatment

Lico is a natural CH extracted from licorice and categorized into five major classes (licoA, licoB, licoC, licoD, and licoE), which have been shown to exhibit antidiabetic, antiallergic, antioxidant, anti-inflammatory, antimicrobial, antiviral, and anticancer activities [21]. All licos possess an OH group on the A and B rings. LicoA and LicoE have a 1,1-dimethyl-2-propenyl” substituent at position C-5. In LicoC and LicoD, there is a prenyl substituent (3-methylbut-2-en-1-yl) at the C-3 and C-3′ positions, respectively (Figure 2). However, the potential mechanisms of these licos in nicotine-induced NSCLC treatment remain unclear. Licos play a significant role in inhibiting the migration, invasion, and proliferation of cancer cells, along with promoting apoptosis and cell cycle arrest through suppressing the activity of α7nAChR-mediated signaling pathways, including PI3K/Akt/mTOR, ERK, and Wnt/β-catenin [55]. LicoE treatment resulted in inhibited cell migration, invasion, metastasis, angiogenesis, and induced apoptosis in mouse lung tissues via upregulating the expression of HIF-1α, CDK, MMP-9, cyclooxygenase-2 (COX-2), vascular endothelial growth factor (VEGF), lymphocyte common antigen 45 (CD45), lymphatic vessel endothelial receptor-1 (LVER-1), and downregulating the expression of Bcl-2-associated X protein (Bax) and cleaved caspase-3 [56]. LicoA, LicoB, and LicoD have been shown to exert anti-inflammatory effects on murine macrophage cell line RAW264.7 by inhibiting α7nAChR-mediated signaling cascades, including lipopolysaccharide (LPS)-induced protein kinase A (PKA), NO production, NF-κB p65 phosphorylation at serine 276, tumor necrosis factor (TNF-α), and monocyte chemoattractant protein-1 (MCP-1) expression [57,58]. Treatment with licoB showed a reduction of oxidative damage in RAW264.7 cells in vivo. LicoB decreases the production of ROS and inflammatory cytokines (e.g., TNFα) and increases the levels of antioxidant substances through the activation of Nrf2 and the downregulation of NF-κB signaling pathways [59]. Therefore, licos may exert anticancer effects in nicotine-induced NSCLC via modulation of the α7nAChR-mediated signaling pathways and/or molecular genes.

Activation of the α7nAChR-mediated signaling pathways, known to be involved in a range of cellular processes, including cell proliferation and anti-apoptosis, was found to increase Aβ levels in NSCLC cells [40]. LicoA and LicoB demonstrated anti-Aβ aggregation activity by collapsing the hydrogen bonds inside the Aβ_1–42_ protofibril to varying degrees [60]. LicoE inhibits Aβ_1–42_ aggregation through suppression of the choline transporter-like protein 1 function in microglia and TNF-α mRNA expression [61]. Thus, licos may inhibit excessive accumulation of Aβ in NSCLC cells, leading to the suppression of α7nAChR and its downstream signaling pathways.

Several in vitro experiments investigating the therapeutic potential of licoA in nicotine-induced NSCLC suggest it exerts several activities, including anti-tumor growth, anti-proliferative, anti-migration, anti-invasion, anti-viability, apoptosis, and autophagy, via modulation of the α7nAChR-mediated signaling pathways and/or molecular genes. Treatment with licoA at a 13 μg/mL concentration showed significant inhibitory effects on the nuclear localization of β-catenin in NSCLC cells by disrupting the activity of Wnt/β-catenin signaling [50]. LicoA treatment demonstrated anti-proliferative and apoptotic effects in NSCLC cells at a concentration of 10 μM through upregualting microRNA (miRNAs) expression, leading to promote endoplasmic reticulum (ER) stress by inhibiting Nrf2 expression. However, treatment with licoA at higher concentrations (in particular 40 μM) enhances autophagy and unfolded protein response (UPR), but without affecting the downstream apoptotic genes of Bim and Bcl-2 in NSCLC cells [62]. LicoA promotes the migration and invasion-inhibiting effects in NSCLC when treated at a concentration of 2–20 μM through the suppression of Sp1 (specificity protein 1) expression and MMP signaling pathways [63]. LicoA suppresses proliferation and promotes apoptosis, autophagy, and cell cycle arrest at the S and G_2_/M phases in NSCLC cells at 40 and 80 μM concentrations through the modulation of apoptotic and anti-apoptotic gene expression [64]. LicoA induces apoptotic and autophagic cytotoxicity in NCI-A549 and NCI-H460 cells at different concentrations by altering the expression of apoptosis and autophagy-related genes through the downregulation of ERK and c-Jun N-terminal kinase (JNK) signaling pathways [65]. LicoA has been reported to significantly repress NSCLC cell viability by inhibiting hypoxia-mediated HIF-1α activation and its related genes at a concentration of 20 μM [66]. It has been demonstrated that licoA inhibits proliferation and promotes apoptosis and cell cycle arrest at the G_2_/M phase at different concentrations by altering cell cycle and apoptosis-related gene expression via the upregulation of the ER stress pathway [67]. LicoA inhibits the growth of gefitinib-resistant NSCLC cells at 50–70 μM concentrations via suppressing heat shock protein 90 (Hsp90) expression, resulting in a significant depletion of oncogenic genes [68]. Treatment of NSCLC cells with licoA at 5–20 μM concentrations led to a significant inhibition of viability and an inducer of apoptosis, autophagy, and cell cycle arrest at the sub-G_1_ phase. The mechanisms behind these effects are associated with increased lactate dehydrogenase (LDH) release and upregulated apoptotic and autophagic-related gene expression [69]. NSCLC cells treated with licoA at a concentration of 10 μM resulted in inhibited proliferation and induced apoptosis by downregulating interferon-gamma (IFN-γ)-induced programmed death-ligand 1 (PD-L1) protein expression through the generation of ROS, the activation of protein kinase RNA-like endoplasmic reticulum kinase-eukaryotic initiation factor 2α (PERK-eIF2α) axis, and the inhibition of eukaryotic translation initiation factor 4E-binding protein 1 (4EBP1) phosphorylation [70].

Treatment with licoA represses cell viability and induces apoptosis in vivo by upregulating apoptosis-related genes and inhibiting tyrosine-protein kinase Met (c-Met) phosphorylation and its downstream kinases-mediated gefitinib resistance in NSCLC cells through promoting c-Casitas B-lineage lymphoma (c-Cbl), which is responsible for licoA-induced c-Met degradation [71]. LicoA treatment at 20 and 40 μM concentrations resulted in a significant inhibition of proliferation in vitro and xenograft tumor growth in vivo, along with the induction of apoptosis and cell cycle arrest at the G_1_ phase. The mechanisms behind these effects in NSCLC cells are related to the upregulation and/or downregulation of molecular genes and signaling pathways involved in cell proliferation and apoptosis [72]. Treatment with licoA inhibits in vitro and in vivo cell growth of osimertinib-sensitive and osimertinib-resistant NSCLC cells at different concentrations, with no significant cytotoxicity against these cells observed. LicoA treatment also induces apoptosis by suppressing survivin protein via the downregulation of EGFR and its downstream kinases [73].

LicoB treatment has shown anticancer effects on gefitinib-sensitive and gefitinib-resistant NSCLC cells in vitro at 5–20 μM concentrations by inducing proliferation inhibition, apoptosis, and cell cycle arrest at the G_2_/M phase. LicoB treatment significantly inhibited EGFR, mesenchymal epithelial transition factor receptor (MET) activity, apoptotic gene expression, and enhanced ROS, ER stress, and pro-apoptotic gene expression [74]. Treatment with licoD at the same concentrations also resulted in suppressed phosphorylation and the kinase activity of EGFR and MET, induced apoptosis, and blocked cell cycle progression at the G_2_/M phase in gefitinib-sensitive and gefitinib-resistant NSCLC cells in vitro [75].

**Figure 4:**
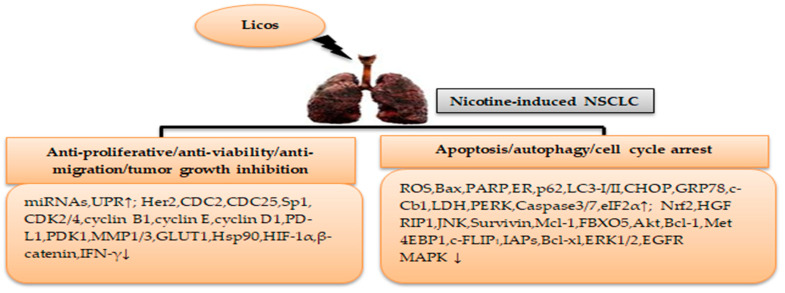
Mechanisms of licos in nicotine-induced NSCLC treatment. (↓) decrease, (↑) increase.

Table 2 summarizes the effects of licorice licos in nicotine-induced NSCLC treatment. Taken together, licoA, licoB, and licoD may be effective in nicotine-induced NSCLC treatment via modulating the α7nAChR-mediated molecular genes and/or cellular signaling pathways (Figure 4).

## 5. Licorice Echinatin in Nicotine-Induced NSCLC Treatment

ECH, a CH isolated from licorice, is structurally similar to licoA, having one OH group on the A and B rings (positions 4′ and 4) (Figure 2). ECH exerts strong antioxidant effects by generating two radical adduct formation products (ECH-ECH dimer and ECH-2-1,1-diphenyl-2-picrylhydrazyl radical adducts) through hydrogen atom transfer antioxidant mechanisms [76]. However, whether ECH has a therapeutic role in nicotine-induced NSCLC remains unknown. Only one in vitro experiment has examined the therapeutic effect of ECH against NSCLC cells. The experiment demonstrated anti-proliferative, apoptotic, and autophagic effects in gefitinib-sensitive and resistant NSCLC cells (NCI-HCC827, NCI-HCC827GR) when treated at a concentration of 5–15 μM by inhibiting the expression of EGFR, MET, Akt, ERK, and increasing ROS production, all known as α7nAChR-mediated molecular genes involved in NSCLC development [77].

## 6. Limitations

Most experiments investigating the anticancer effects of licorice CHs in nicotine-induced NSCLC have been performed in vitro, with only a few in vivo mouse models being used. Licorice CHs have never been assessed for their clinical significance, and there are no tools for monitoring the effectiveness and safety of these CHs in NSCLC patients. The extraction and identification of CHs have not been clearly determined. A few in vitro experiments showed high cytotoxicity of ILTG and licoA treatment, which could be useful in controlling the growth of NSCLC cells by enhancing apoptosis and autophagy in these cells. All experiments showed dose-dependent anticancer effects, but the optimal dose in treatment remains a key challenge. Licorice CHs have not been assessed for their bioavailability in all experiments.

## 7. Conclusions

This review highlights the effects of licorice CHs in nicotine-induced NSCLC treatment via various mechanisms of action. ILTG, licoA–E, and ECH are natural CHs isolated from licorice with anticancer effects in vitro and/or in vivo. However, the mechanisms of licorice CHs in nicotine-induced NSCLC treatment remain largely unknown. ILTG may produce anticancer effects in nicotine-induced NSCLC by PAM of α7nAChR. ILTG may also act as a potential inhibitory compound for MAO-induced cellular processes in NSCLC cells through the suppression of α7nAChR-mediated signaling pathways. ILTG and licos may have a significant role in repressing Aβ aggregation in NSCLC cells, leading to inhibited α7nAChR and its downstream signaling pathways implicated in NSCLC development.

In vitro and/or in vivo experiments have shown that licorice CHs exert multiple therapeutic effects in nicotine-induced NSCLC, including anti-proliferative, anti-tumor growth, anti-invasion, anti-migration, anti-viability, apoptosis, cell cycle arrest, and autophagy through modulating α7nAChR-mediated signaling pathways and molecular genes. Treatment with ILTG, licoA, licoB, and licoD has demonstrated anticancer effects on TKI, osimertinib, and gefitinib-sensitive and resistant NSCLC cells by inducing apoptosis and/or cell cycle arrest via upregulating apoptosis-related genes.

Although all experiments demonstrated that they were effective in NSCLC treatment, the exact mechanisms involved in the therapeutic effects of licorice CHs against NSCLC cells induced by nicotine as a stimulator of α7nAChR need further investigation. More in vitro and in vivo experiments are warranted to evaluate the toxicity, safety, and optimal effective dose of licorice CHs in nicotine-induced NSCLC treatment. Further clinical studies are needed to investigate the therapeutic role of licorice CHs in nicotine-induced NSCLC, particularly in smokers.

## Figures and Tables

**Table 1:** Therapeutic effects of licorice ILTG in nicotine-induced NSCLC.

Experimental Models	NSCLC Cell Line	Dose/Administration	Therapeutic Effects	Ref.
In vitro	A549	A549 cells were treated with ILTG at 20 μM concentration for 24–72 h incubation at 37 °C	Anti-proliferative, anti-invasion, anti-migration, apoptosis	[46]
In vitro	A549	A549 cells were treated with ILTG at 0, 2, 10, 20, and 40 μmol/L concentrations for 12–72 h of incubation at 37 °C	Anti-proliferative, apoptosis, G_1_ phase cell cycle arrest	[47]
In vitro	A549	A549 cells were treated with ILTG at 10, 20, 30, 40, and 40 μM concentrations for 24–72 h of incubation at 42 and 70 °C	Anti-proliferative, G_2_/M phase cell cycle arrest	[48]
In vitro	A549	A549 cells were treated with ILTG and flavonoid glycosides (liquiritin, isoliquiritin) at 1.95–500 μg/mL concentrations for 24–72 h of incubation	Apoptosis, G_2_/M phase cell cycle arrest	[49]
In vitro	SK-LU-1	SK-LU-1cells were treated with ILTG at 1.56, 3.13, 6.25, 12.5, 25, and 50 μg/mL concentrations for 24–72 h of incubation	Inhibition of cell growth	[50]
In vitro	A549, H1299	NSCLC cells were treated with ILTG at 6.25, 12.5, and 25 μM concentrations for 24–72 h of incubation at 37 °C	Anti-proliferative, anti-invasion, anti-migration	[51]
In vitro	A549	A549 cells were treated with pure ILTG and ILTG nanosuspensions at 0.03, 0.06, 0.09, 0.12, 0.15, and 0.15 μM concentrations for 24–72 h incubation at 37 °C	Apoptosis	[52]
In vitro/vivo	A549, H1299, H1975	NSCLC cells were treated with ILTG at 0, 3, and 10 μM concentrations for 24 h of incubation at 37 °C Athymic nude mice were injected with a mixture of DMEM and Matrigel in a 1:1 ratio and then classified into 4 groups, in which each group received a vehicle: ILTG (25 mg/kg), tetrahydroxychalcone (25 mg/kg), or AZD0530 (20 mg/kg)	Anti-migration, inhibition of cell growth, and tumorigenesis	[53]
In vitro/vivo	A549, HCC827GR, H1975, H1650, and HCC827	NSCLC cells were treated with ILTG at 0, 10, 20, and 40 μM concentrations for 24 h of incubation at 37 °C Athymic nude mice were divided into 4 groups: vehicle, ILTG (1 mg/kg), ILTG (5 mg/kg), and gefitinib (5 mg/kg)	Apoptosis	[54]

**Table 2:** The therapeutic effects of licorice licos in nicotine-induced NSCLC.

Experimental Models	Lico Classification	NSCLC Cell Line	Dose/Administration	Therapeutic Effects	Ref.
In vitro	LicoA	SK-LU-1	SK-LU-1cells were treated with licoA at 1.56, 3.13, 6.25, 12.5, 25, and 50 μg/mL concentrations for 24–72 h of incubation	Inhibition of tumor growth	[50]
In vitro	LicoA	H292	H292 cells were treated with licoA at 0, 1, 10, 20, 40, and 80 μM concentrations for 24–96 h of incubation at 37 °C	Anti-proliferative, apoptosis, and autophagy	[62]
In vitro	LicoA	A549, H460	NSCLC cells were treated with licoA at 0, 2, 5, 10, and 20 μM concentrations for 24 and 48 h of incubation at 37 °C	Anti-migration, anti-invasion	[63]
In vitro	LicoA	A549	A549 cells were treated with licoA at 20, 40, and 80 μM concentrations for 24 h of incubation at 37 °C	Apoptosis, S and G_2_/M phase cell cycle arrest, autophagy	[64]
In vitro	LicoA	A549, H23, H460, H1299, SPC-A1	NSCLC cells were treated with licoA at 0, 5, 10, 20, 30, and 40 μM concentrations for 48 h of incubation at 37 °C for 7 days	Apoptosis, autophagy	[65]
In vitro	LicoA	HCT116, H1299, H322	NSCLC cells were treated with licoA at 5, 10, 15, and 20 μM concentrations for 6 h of incubation at 37 °C	Anti-viability	[66]
In vitro	LicoA	A549, H460	NSCLC cells were treated with licoA at 20, 40, and 60 μM concentrations for 4 and 12 h of incubation at 37 °C	Anti-proliferative, apoptosis, G_2_/M phase cell cycle arrest	[67]
In vitro	LicoA	H1975	H1975 cells were treated with licoA at 0, 10, 30, 50, and 70 μM concentrations for 24–72 h of incubation at 37 °C	Anti-proliferative	[68]
In vitro	LicoA	A549, H1299	NSCLC cells were treated with licoA at 0, 5, 10, 15, and 20 μM concentrations for 24 h of incubation at 37 °C	Apoptosis, sub-G_1_ phase cell cycle arrest, autophagy	[69]
In vitro	LicoA	A549, H1299, H1650	NSCLC cells were treated with licoA at 5, 10, 15, and 20 μM concentrations for 24 and 48 h of incubation at 37 °C	Anti-proliferative, apoptosis	[70]
In vivo	LicoA	HCC827, PC-9	Female athymic nude mice were injected with 20 mg/kg licoA for a period of six weeks	Anti-viability, apoptosis	[71]
In vitro/vivo	LicoA	H226, H1703	NSCLC cells were treated with licoA at 0, 10, 20, and 40 μM concentrations for 24–72 h incubation at 37 °C BALB/c-nu mice (male) were divided into 4 groups: control (saline containing 20% SBE-β-CD), licoA (7.5 mg/kg), licoA (15 mg/kg), and cisplatin (2 mg/kg)	Anti-proliferative, inhibition of tumor growth, apoptosis, G_1_ phase cell cycle arrest	[72]
In vitro/vivo	LicoA	A549, HCC827, H1975, and H3255	NSCLC cells were treated with licoA at 0, 5, 10, 20, 40, 80, and 200 μM concentrations for 24–96 h of incubation at 37 °C Athymic nude mice (female) were injected with 10 mg/kg licoA	Inhibition tumor growth, apoptosis	[73]
In vitro	LicoB	HCC827, HCC827GR	NSCLC cells were treated with licoB at 5, 10, 15, and 20 μM concentrations for 48 h of incubation at 37 °C	Anti-proliferative, apoptosis, G_2_/M phase cell cycle arrest	[74]
In vitro	LicoD	HCC827, HCC827GR	NSCLC cells were treated with licoD at 5, 10, 15, and 20 μM concentrations for 48 h of incubation at 37 °C	Anti-proliferative, apoptosis, G_2_/M phase cell cycle arrest	[75]

## Abbreviations

4EBP1	Eukaryotic translation initiation factor 4E-binding protein 1
Akt	Threonine kinase
Aβ	Amyloid-beta
Bax	Bcl-2-associated X protein
Bcl-2	B-cell lymphoma-2
Bcl-xl	B-cell lymphoma-extra large
Bim	B-cell chronic lymphocytic leukemia-lymphoma like 11 gene
c-Cbl	c-Casitas B-lineage lymphoma
CD45	Lymphocyte common antigen 45
CDC2	Cell division control protein 2 homologue
CDC25	Cell division control protein 25 homologue
CDC42	Cell division control protein 42 homologue
CDK	Cyclin-dependent kinase
c-FLIP_L_	Cellular-FLICE inhibitory protein
CHOP	C/EBP homologous protein
CHs	Chalcones
c-Met	Tyrosine-protein kinase Met
COX-2	Cyclooxygenase-2
CREP	cAMP-responsive element-binding protein
CS	Cigarette smoke
DMEM	Dulbecco’s Modified Eagle Medium
ECH	Echinatin
EGFR	Epidermal growth factor receptor
EGR1	Early growth response 1
eIF2α	Eukaryotic initiation factor 2α
EMT	Epithelial-mesenchymal transition
ER	Endoplasmic reticulum
ERK	Extracellular signal-regulated kinase
FAK	Focal adhesion kinase
FBXO5	F-box protein 5
GADD45	Growth Arrest and DNA Damage-inducible 45
GLUT1	Glucose transporter 1
GRP78	78-kDa glucose-regulated protein
GSK	Glycogen synthase kinase
GTPase	Guanosine triphosphatase
Her2	Human epidermal growth factor receptor 2
HGF	Hepatocyte growth factor
HIF-1α	Hypoxia inducible factor-1
HPV	Human papillomavirus
Hsp90	Heat shock protein 90
IAPs	Inhibitor of apoptosis proteins
IFN-γ	Interferon-gamma
IGF2BP3	Oncofetal IGF2 mRNA-binding protein 3
ILTG	Isoliquiritigenin
JNK	c-Jun N-terminal kinase
LC	Lung cancer
LC3	LC3-phospholipid conjugate
LDH	Lactate dehydrogenase
LEVR-1	Lymphatic vessel endothelial receptor-1
Licos	Licochalcones
LPS	Lipopolysaccharide
MAO	Monoamine oxidase
MAPK	Mitogen-activated protein kinase
MCP-1	Monocyte chemoattractant protein-1
MDM2	Murine double minute 2
Met	Mesenchymal epithelial transition factor receptor
mFasL	Membrane-bound Fas ligand
miRNAs	MicroRNAs
MMP	Metalloproteinase
mTOR	Mammalian target of rapamycin
NF-kB	Nuclear transcription factor-kappaB
NNK	4-(Methylnitrosamino)-1-(3-pyridyl)-1-butanone
NNN	*N*-Nitrosonornicotine
NO	Nitric oxide
Nrf2	Nuclear factor erythroid-2 related factor 2
NSCLC	Non-small cell lung cancer
OH	Hydroxyl group
p21	Protein 21
p53	Protein 53
p62	Protein 62
p65	Protein 65
p70	Protein 70
PAMs	Positive allosteric modulators
PARP	Poly ADP ribose polymerase
PCNA	Proliferating cell nuclear antigen
PDK1	Pyruvate dehydrogenase kinase 1
PD-L1	Programmed death-ligand 1
PERK	Protein kinase RNA-like endoplasmic reticulum kinase
PI3K	Phosphatidylinositol-3 kinase
PKA	Protein kinase A
PPARγ	Peroxisome proliferator-activated receptor γ
PTEN	Phosphatase and tensin homolog
Raf	Retinoblastoma tumor suppressor protein-proto-oncogene
RIP1	Receptor-interacting protein-1
ROS	Reactive oxygen species
SCLC	Small cell lung cancer
sFasL	Soluble Fas ligand
Sp1	Specificity protein 1
Src	Non-receptor tyrosine kinase
STAT	Signal transducer and activator of transcription
TKI	Tyrosine kinase inhibitor
TNFα	Tumor necrosis factor
TWIST1	Twist family bHLH transcription factor 1
Tyr	Tyrosine
UPR	Unfolded protein response
VEGF	Vascular endothelial growth factor
WAF1/CIP1	Cyclin-dependent kinase inhibitor p21
α7nAChR	Nicotinic acetylcholine receptor
β-AdrR	beta-adrenergic receptor
